# Envelope Extraction for Vibration Locating in Coherent Φ-OTDR

**DOI:** 10.3390/s22031197

**Published:** 2022-02-04

**Authors:** Wei Zan, Yu Wang, Xin Liu, Pengfei Wang, Baoquan Jin

**Affiliations:** 1 Key Laboratory of Advanced Transducers and Intelligent Control Systems (Ministry of Education and Shanxi Province), College of Physics and Optoelectronics, Taiyuan University of Technology, Taiyuan 030024, China; zanwei234@163.com (W.Z.); wangyu@tyut.edu.cn (Y.W.); 2 College of Information and Computer, Taiyuan University of Technology, Taiyuan 030024, China; liuxin01@tyut.edu.cn; 3 College of Electrical and Power Engineering, Taiyuan University of Technology, Taiyuan 030024, China; w159753wpf@163.com

**Keywords:** phase-sensitive optical time-domain reflectometry, envelope extraction, vibration locating, beating signal

## Abstract

In a coherent phase-sensitive optical time-domain reflectometry (Φ-OTDR) sensing system, a frequency shift of hundreds of MHz generated by the pulse modulation of an acoustic optic modulator results in a high central frequency of a beating signal spectrum. In order to reduce the high-performance hardware requirement of signal acquisition, the coherent Φ-OTDR based on envelope extraction is proposed in this paper. Firstly, a theoretical model of a quasi-sinusoidal amplitude-modulated signal is built for the beating signal between local oscillator light and Rayleigh backward scattering light. An envelope detector is then utilized to realize the envelope extraction of beating signals with advantages of a simple structure and quick response. The extracted envelope can be directly used for vibration locating without the conventional orthogonal demodulation. Experiment results present that the sampling rate can be reduced to 10 MHz under the spatial resolution of 10 m and the sensing distance of 31 km. This scheme proves that envelope extraction is a reliable technical route for vibration locating, which can effectively reduce the sampling rate and simplify the data demodulation.

## 1. Introduction

Phase-sensitive optical time-domain reflectometry (Φ-OTDR) has the advantages of resistance to electromagnetic interference, high insulation strength, and corrosion resistance. It is a fully distributed sensor with the ability to locate external vibration over a long distance [1,2]. This sensing system has good prospects in perimeter security, power line monitoring, gas pipeline monitoring, railway system monitoring, and geological and hydrological monitoring [3,4,5,6,7].

Direct detection Φ-OTDR is firstly proposed to detect the intensity of a Rayleigh backscattered (RBS) signal [8]. RBS curves appear in a jagged form due to the fluctuation of optical intensity and phase, mainly caused by the inhomogeneous spatial distribution of the fiber refractive index and external disturbance [9]. The direct detection sensing system is used to locate vibration events according to the intensity differences among the RBS traces over time. However, the low RBS signal intensity limits the performance enhancement of direct detection Φ-OTDR. A Raman amplifier could compensate for the RBS signal intensity; however, it increases the cost and complexity [10]. Therefore, Healey and Malyon proposed the method of coherent detection to improve the optical intensity of an RBS signal [11]. Furthermore, the coherent detection scheme has been adopted in the Φ-OTDR system to obtain a higher signal-to-noise ratio (SNR) [12].

However, a high-frequency shift is required to ensure high spatial resolution and spectrum distortionless in coherent Φ-OTDR. An acoustic optic modulator (AOM) is normally used to generate the frequency shift of hundreds of MHz and to realize the pulse modulation from continuous light. It results in a high central frequency of the beating signal spectrum in the coherent detection scheme. Therefore, a sampling rate up to GSps is often needed in coherent Φ-OTDR to entirely obtain the beating signals [13]. This extra-high hardware requirement of signal acquisition leads to data analysis challenges and increases system costs [14]. So, there is a need for a cost-efficient scheme with a low sampling rate and acquisition bandwidth.

To date, many research works are proposed to reduce the sampling requirement in coherent Φ-OTDR. Soriano-Amat and Miguel et al. proposed an approach that allows a customized time expansion, this technique reaches cm-scale spatial resolutions over 1 km while requiring a remarkably low detection bandwidth in the MHz regime [15]. X. P. Zhang et al. use the undersampling theory to reduce the data volume of digital I/Q demodulation. This work reduces the sampling rate to 71 MSps. However, the SNR deteriorates due to spectrum aliasing during undersampling [16]. They also use a 3 dB quadrature hybrid coupler directly on the beating signal and acquire the analog in-phase and the quadrature components later. A two-channel data acquisition card (DAQ) with a sampling rate of 100 MSps is used to sample the I/Q signals [17]. This scheme needs to meet the constraint conditions, such as a perfect 90° phase difference after the coupler and amplitude balance between I and Q signal. These above methods promoted the development of coherent Φ-OTDR, which remain a noticeable sampling cost.

In order to reduce the high-performance hardware requirement of signal acquisition, this paper proposes a low sampling rate demodulation scheme based on envelope extraction for coherent Φ-OTDR using an envelope detector. This scheme is realized by extracting the upper-envelope signal, avoiding the direct processing of high-frequency beating signals in traditional coherent Φ-OTDR. Thus, orthogonal demodulation and Hilbert-–Huang transformation are unnecessary for these situations aiming at vibration locating. This scheme provides different approaches to decrease hardware costs and calculation amounts. The experimental results are demonstrated, showing that the scheme with low sampling requirements has a similar performance compared with the I/Q demodulation algorithm during vibration locating.

## 2. Theoretical Analysis and Principles

### 2.1. Backscattering Model of Coherent Φ-OTDR

In a coherent Φ-OTDR system, a laser source with narrow linewidth is used for emitting a continuous-wave laser, an arbitrary waveform generator (AWG) generates a series of pulses to trigger the process of data acquisition and pulse modulation. Regularly, an AOM is applied to create interrogation pulses. The amplified interrogation pulses are injected into the optical fiber through the circulator. The laser with narrow linewidth enables the high coherence of RBS light, for the RBS remains almost an unchanged wavelength of the input pulse light in a short time. Meanwhile, the RBS light is output through the circulator and received by the photodetector (PD) [18]. The generated photoelectric current is amplified by a trans-impedance amplifier, following an analog-to-digital conversion after PD. Finally, the RBS curve is obtained by a DAQ.

**Figure 1:**
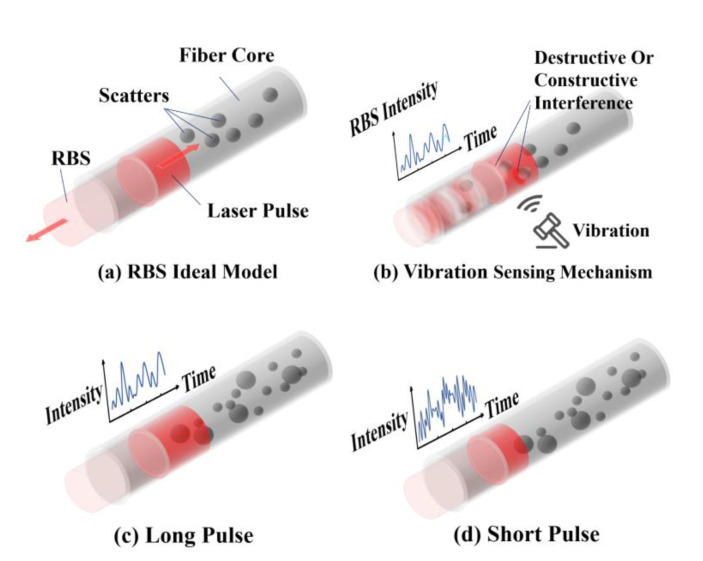
Sensing mechanism of Φ-OTDR based on RBS equivalent model. (**a**) Backscattering model; (**b**) vibration sensing mechanism of Φ-OTDR; (**c**,**d**) RBS curves with varying pulse width.

According to the scattering model, The backscattering process is modeled by a set of separate scatterers whose amplitude is characterized as statistically independent random Gaussian variables [19], and the phase follows a uniform distribution [20,21]. Therefore, it can be equivalent to separate scattering points in the fiber core, which is shown in Figure 1a.

As shown in Figure 1b, the interference of previously generated backscattered light and the currently generated backscattered light is realized within the pulse. The RBS curve appears as a stochastic shape for the fluctuation of the refractive index along with the fiber. According to Hooke’s law and photoelastic effect, any external perturbations change light path *z* by introducing refractive index Δ*n* and local shape difference Δ*l* in Equation (1). This explains the causes of the varying state of constructive interference and destructive interference in Figure 1b.

(1) $$\begin{array}{ll} \Delta z_{path} & =\left(n_{0}+\Delta n\right)\left(l+\Delta l\right)-n_{0}l\\ \Delta n & =e_{z}\xi n_{0}\\ \Delta l & =e_{z}\eta l \end{array}$$

In Equation (1), l is the length of the optical fiber, $n_{0}$ is the refractive index of the optical fiber, η is the elastic coefficient, ξ is photoelastic coefficient, and $e_{z}$ is the axial strain. The phase variations can be represented as:

(2) $$\Delta \Phi =\frac{2\pi \Delta z_{path}}{\lambda }$$

In Equation (2), λ is the wavelength of the light. The electric field of the RBS is represented as:

(3) $$\begin{array}{ll} E_{R} & =E_{0}\overset{N}{\underset{i=1}{\sum }}rect\left(z-z_{i}\right)e^{-2\alpha z_{i}}e^{j2\pi \omega_{0}t+j2\pi \omega t}e^{r_{i}+j\Delta \varphi_{i}+\Delta r_{i}+j\Delta \phi_{i}}\\ rect\left(x\right) & =\{\begin{array}{c} 0,\;else\;where\\ 1,\;\;\left|x\right|<\frac{n_{0}l}{2N} \end{array} \end{array}$$

In Equation (3), $E_{R}$ is the scattering light amplitude, $E_{0}$ is the initial amplitude of the RBS light, rect(z) is a rectangle function, *N* is the number of discrete points, the loss coefficient α is 0.20 dB/km at the wavelength of 1550 nm, $\omega_{0}$ is the angular frequency of the pulsed light, ω is the modulation frequency of the AOM, and $r_{i}+j\Delta \varphi_{i}$ is the random intensity and phase modulation caused by the scatters and interference effect within the pulse. It indicates the essential characteristics of the optical fiber. $\Delta r_{i}+j\Delta \phi_{i}$ is the intensity and phase change caused by external disturbance at the distance of $z_{i}$.

With the moving differential algorithm applied to the RBS traces, the result is shown as Equation (4).

(4) $$\Delta E_{R\left(z=z_{i}\right)}=E_{0}e^{-2\alpha z_{i}}e^{j2\pi (\omega_{0}+\omega )t+j\Delta \varphi_{i}+j\Delta \phi_{i}}e^{r_{i}+\Delta r_{i}}$$

In Equation (4), $\Delta \varphi_{i}$ is a negligible and slowly varying quantity caused by the frequency drift of the laser. In addition, the laser phase noise could also lead to the instability of the results [22]. $\omega_{0}$ represents the laser central frequency which reaches 193.5 THz at 1550 nm, the term $\omega_{0}+\omega$ far exceeds the bandwidth of the acquisition system. This means that the term $e^{j2\pi \omega_{0}t+j2\pi \omega t}e^{j\Delta \varphi_{i}+j\Delta \phi_{i}}$ can be regarded as a constant with this bandwidth limited system.

Combining the slowly changing parts as $R_{i}$, the Equation is simplified as (5).

(5) $$\Delta E_{R\left(z=z_{i}\right)}=R_{i}e^{\Delta r_{i}}$$

where $R_{i}$ is the amplitude of the backward light reflected by the *i*th virtual scatter. In Equation (5), the result of differential progressing is directly proportional to the $R_{i}$ and $e^{\Delta r_{i}}$. By discriminating the intensity difference $e^{\Delta r_{i}}$, the external vibration could be detected.

The backscattered light amplitudes of most areas along with the optical fiber are stable and unchanged in the absence of vibration. The low—frequency drift of the laser and other thermal noise mainly cause these slight changes [19]. On the contrary, high-frequency components appear when the phase changes according to external vibration. This can be used for vibration locating.

The accurate vibration locating is based on narrow pulse width, enough signal bandwidth and acquisition bandwidth. Comparing Figure 1c and Figure 1d, the narrower the pulse injected into the optical fiber, the more information can be obtained from RBS curves. It means there is a higher ability to distinguish adjacent vibration events because more separate points are used for describing the same length, which can be presented as a larger N in Equation (3). It is possible to describe optical fiber states in a more precise way.

For the ideal experiment condition, the limited bandwidth leads to a low noise level with acceptive spatial resolution reduction [13]. Every segment, before analyzing the RBS signal, may cause the loss of useful information. It is summarized into three critical indexes: pulse width, PD bandwidth, and the sampling rate of the DAQ. Therefore, the system’s spatial resolution is determined by the least one among these limits. The spatial resolution Δz of the vibration sensor is determined by the minimum of the response bandwidth of the acquisition system, the sampling rate related to N and the pulse width *T_p_* gated into the fiber, specified as (6).

(6) $$\Delta z=cT_{p}/2n_{g}$$

where *c* is the speed of light in a vacuum, and $n_{g}$ is the group refractive index. Taking an example of a spatial resolution z of 10 m, it corresponds to the pulse width $T_{p}$ of 100 ns. The bandwidth of at least a 10 MHz (1/100 ns) electrical signal needs to be guaranteed. The sampling rate should be more than 10 MSps, allowing the spatial resolution of 10 m at least. Typically, the sampling interval is set to a length no longer than the spatial resolution limited by the pulse width of the probe light [23].

**Figure 2:**
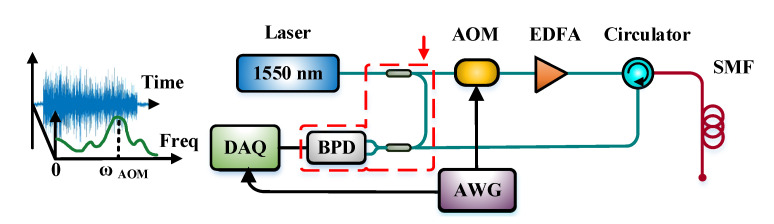
Coherent Φ-OTDR and its typical time-frequency waveform diagram.

Since the sensing performance is limited seriously by the RBS intensity, the coherent detection Φ-OTDR based on the beating signal is proposed. The coherent Φ-OTDR system is shown in Figure 2.

For coherent detection, a local oscillator light is mixed with RBS light through a 2 × 2 coupler, and the optical signal is transformed into an electrical signal with a balanced photodetector (BPD). The collected beating signal within the detection bandwidth is expressed in Equation (7).

(7) $$E_{beat}\propto 2E_{LO}E_{R}\cos\theta \cos\left(\omega t+\Delta \varphi +\Delta \phi \right)$$

where $E_{beat}$ is the amplitude of the beating signal of mixed light, $E_{LO}$ is the amplitude of local light, θ is the polarization angle of two polarization states, assumed stable and ignored in this paper, ω is the angle frequency difference between two light waves, Δφ is the phase difference caused by the scatters, and Δϕ is the phase difference caused by external perturbations.

In the I/Q demodulation algorithm, I and Q mean the in-phase and the quadrature components of the electrical signal, respectively [14]. The band-filtered beating signal is multiplied with a pair of same frequency quadrature quantity cos(ωt) and sin(ωt), respectively. I and Q are obtained as Equations (8) and (9).

(8) $$\begin{array}{l} I\propto E_{LO}E_{R}\cos\left(\omega t+\Delta \varphi +\Delta \phi \right)\cos\left(\omega t\right)\\ \propto E_{LO}E_{R}[\cos\left(2\omega t+\Delta \varphi +\Delta \phi \right)+\cos\left(\Delta \varphi +\Delta \phi \right)] \end{array}$$

(9) $$\begin{array}{l} Q\propto E_{LO}E_{R}\cos\left(\omega t+\Delta \varphi +\Delta \phi \right)\sin\left(\omega t\right)\\ \propto E_{LO}E_{R}[\cos\left(2\omega t+\Delta \varphi +\Delta \phi \right)-\sin\left(\Delta \varphi +\Delta \phi \right)] \end{array}$$

For Equations (8) and (9), the results I and Q are filtered by a low-pass filter to remove the quadratic component of cos(2ωt+Δφ+Δϕ). I and Q components are then combined to get the amplitude of RBS $E_{R}$, shown in Equation (10).

(10) $$E_{R}\propto \sqrt{I^{2}+Q^{2}}$$

Similar to the technique applied in the direct detection scheme in Equation (5), the moving differential algorithm is also suitable to locate the area influenced by external perturbations in coherent Φ-OTDR.

Assuming one RBS signal with a bandwidth frequency of Ω in the spectrum, the RBS signal could be replaced with a sinusoidal signal at the frequency of Ω in Equation (7). After ignoring the slowly changing component in a static optical fiber, we get Equation (11).

(11) $$E_{beat}\propto E_{R}\cos\left(\Omega t\right)\cos\left(\omega t\right)$$

Equation (11) shows that the beating signal has the same form as a quasi-sinusoidal amplitude-modulated (AM) signal. Where cos(ωt) is the carrier signal, $E_{R}\cos\left(\Omega t\right)$ is the simplified original signal which needs to be demodulated. This approximation implies the feasibility of getting an RBS amplitude based on AM demodulation methods, such as the envelope extraction scheme.

Based on the analysis of amplitude information from the beating signal, vibration locating demodulation can be accomplished without the phase information in coherent Φ-OTDR system. The envelope of the beating signal could be a substitute for the amplitude result of I/Q demodulation or the intensity of the amplified RBS signal.

### 2.2. Demodulation Principle of Envelope Extraction

It is common to build a coherent Φ-OTDR system with a high sampling rate of the acquisition system and an ultra-performance computing platform for conventional digital I/Q demodulation. As expressed in Equation (11), an AM signal can replace the modulated RBS signal. For the typical demodulation process of an AM signal, filtering out the carrier frequency cos(ωt) and removing high-frequency components by a low-pass filter is indispensable. The envelope of the modulated signal is equivalent to the baseband signal [24].

**Figure 3:**
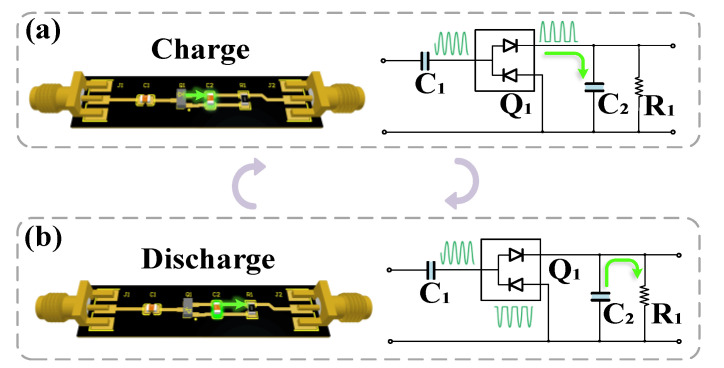
Circuit model of envelope detector. (**a**) The process of charging C_2_; (**b**) the process of discharge from C_2_ to R_1_.

There are many analog and digital methods to realize envelope extraction, such as RMS-to-DC conversion, the lock-in amplifier, peak-hold method (peak detector), coherent demodulator, Lyapunov filter, and Kalman filter [25]. In the application to extract an RBS signal from a single-wave carrier near ω (the frequency shift of AOM) the peak detector method can yield a fast estimate with low latency in the analog domain, implemented by simple hardware circuits, as shown in Figure 3.

In the circuit shown in Figure 3, a circuit model of an envelope detector is regarded as a combination of a rectifier and lowpass filter. Based on this assumption, Q_1_ is an ideal zero bias Schottky detector diode. As shown in Figure 3a, the input AC signal at the left end acts on the diodes Q_1_ in a positive direction, the current flows into the ground through resistance R_1_, and the voltage at both ends of C_2_ charges the capacitor. As shown in Figure 3b, the reverse voltage acts on the diodes Q_1_, the current cannot pass through to the right part, the charged capacitor C_2_ begins to discharge. The discharge speed is related to the time constant $\tau_{2}$, which equals R_1_ * C_2_. When $\tau_{2}$ is much greater than the reciprocal of the high-frequency as the shortest period of the signal, the high-frequency component would be filtered out.

Using the unidirectional conductivity of the diode makes the circuit charge and discharge time constants different. There is a considerable difference between two time constants. Therefore, the choice of time constants is essential.

An ideal modulated signal can be expressed as u=V(1+mcosΩt)cosωt. V and m stand for the amplified coefficient, the constant value of 1 stands for the DC component of the original signal, ω stands for the frequency of the carrier, and Ω denotes the angle frequency of the modulated signal.

Based on these settings, we can find the limits to get the time constants of the RC circuit. The charge time constant is $\tau_{1}=r_{d}*\;$C_2_. $r_{d}$ is the dynamic resister of the surface mount zero bias Schottky detector diodes used in the circuit. It can be measured as a smaller value typically. The discharge time constant $\tau_{2}$ equals to the product of R_1_ and C_2_. To achieve the goal that the demodulated envelope could reflect the original signal, these two conditions need to be met:

(12) $$R_{1}C_{2}\ll \frac{1}{\omega }$$

(13) $$R_{1}C_{2}\gg \frac{1}{\Omega }$$

These conditions can ensure the effect that the voltage change rate at both ends of the capacitor is much greater than that of the envelope. Moreover, it is much less than that of the high-frequency carrier. The inertia distortion phenomenon may appear when the capacitance value of C_2_ is unreasonable in the envelope detector.

The input AM signal can be expressed as (14):

(14) u=V(1+mcosΩt)cosωt

The process of envelope extraction is approximated by a second-order Taylor expansion nonlinear system, and its output can be expressed as:

(15) $$i=u_{0}+\frac{u^{\prime }}{2!}u+\frac{u^{\prime \prime }}{3!}u^{2}\;=a_{0}+a_{1}u+a_{2}u^{2}$$

Equation (15) can be simplified by the substitution process, shown as:

(16) $$i_{0}=a_{0}+a_{1}V\left(1+m\cos\Omega t\right)\cos\omega t+a_{2}{\left[V\left(1+m\cos\Omega t\right)\cos\omega t\right]}^{2}$$

(17) $$\begin{array}{ll} i_{0} & =a_{0}+\frac{1}{2}a_{2}V^{2}\left(1+\frac{1}{2}m^{2}\right)+a_{2}V^{2}\left(m\cos\Omega t+\frac{m^{2}}{4}\cos2\Omega t\right)\\ & +a_{1}V\left[\cos\omega t+\frac{m}{2}\cos\left(\omega +\Omega \right)t+\frac{m}{2}\cos\left(\omega -\Omega \right)t\right]\\ & +\frac{1}{2}a_{2}V^{2}[\left(1+\frac{m^{2}}{2}\right)\cos2\omega t+m\cos\left(2\omega +\Omega \right)t+m\cos\left(2\omega -\Omega \right)t\\ & +\frac{m^{2}}{4}\cos2\left(\omega +\Omega \right)t+\frac{m^{2}}{4}\cos2\left(\omega -\Omega \right)t] \end{array}$$

A low-pass filter is applied as the frequency selection network. The filter cutoff frequency is designed to eliminate the high-frequency carrier but maintains the envelope signal with low-frequency. Therefore, ignore the polynomials containing the factors cosωt, cos2ωt, cos(ω±Ω)t, cos(2ω±Ω)t and cos2(ω±Ω)t, as shown in Equation (18).

(18) $$i_{0}=a_{0}+\frac{1}{2}V^{2}\left(1+\frac{m^{2}}{2}\right)+a_{2}V^{2}\left(m\cos\Omega t+\frac{m^{2}}{4}\cos2\Omega t\right)$$

After the DC component isolating circuit, the original modulated signal at the frequency of Ω in the AM signal is restored, completing the process of envelope extraction.

## 3. System Description and Implementation

### 3.1. Experimental Setup

**Figure 4:**
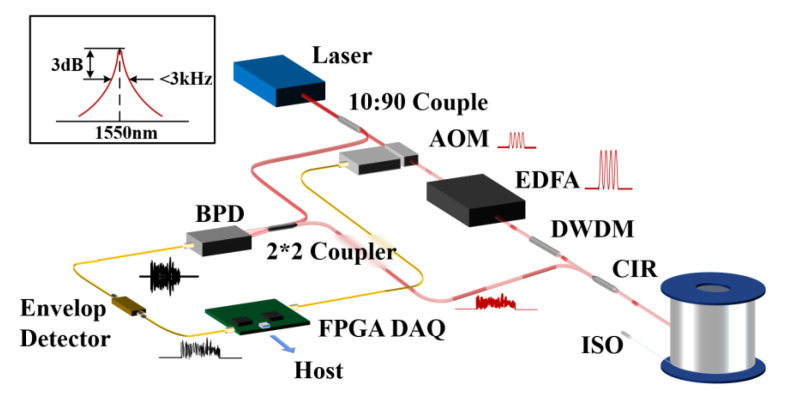
Experimental setup of Coherent Φ-OTDR Sensing System.

The experiment setup is illustrated in Figure 4 based on the principle of coherent Φ-OTDR. A narrow linewidth laser with a linewidth of 3 kHz is used, and its center wavelength is 1550 nm. The average output power of the laser is set to 15 mW. Then, the light is divided into two paths with a 90:10 coupler. The 90% light is modulated into a pulsed light by an AOM with the frequency shift of 80 MHz or 200 MHz alternatively. The 10% light is regarded as a local oscillator light to be mixed with the RBS light later. The FPGA module sends a series of trigger signals and makes the AOM modulate the continuous light into a pulsed light. The pulse repetition frequency is 8 kHz, and the pulse width is 100 ns, corresponding to the spatial resolution of 10 m. The pulsed light is injected into an EDFA to amplify the inferior pulse light, whose average power is amplified to about 1 mW measured by an optical power meter. Next, the pulsed light goes through a dense wavelength division multiplexer (DWDM), which can filter out part of the light of other wavelengths, except 1550 nm, to constrain spontaneous emission noise roused by EDFA. The 1550 nm upper-frequency shifted pulsed light is launched into the sensing fiber via an optical circulator (OC). The interrogation pulse goes through port 1 and out from port 2, then backscattering spin-out from port 3. A piezoelectric transducer (PZT) is applied to simulate the sinusoidal vibration signal at 1 kHz.

The difference between this scheme and the traditional coherent Φ-OTDR structure is that, a high-speed DAQ is necessary to collect the beating signal at the frequency of the carrier band for the I/Q demodulation scheme. In comparison, an upper envelope detector converts the high-frequency alternating signal into a low-frequency signal of tens of megahertz. The frequency of the output signal is greatly reduced, a low-cost DAQ can be used to realize signal collection for the following procedures on the host computer.

### 3.2. System Testing and Preliminary Analysis of Envelope Signals

Before using the system for vibration locating, it is necessary to confirm the high availability of the envelope signal and its accuracy. So, the waveform and spatial resolution should be firstly analyzed.

**Figure 5:**
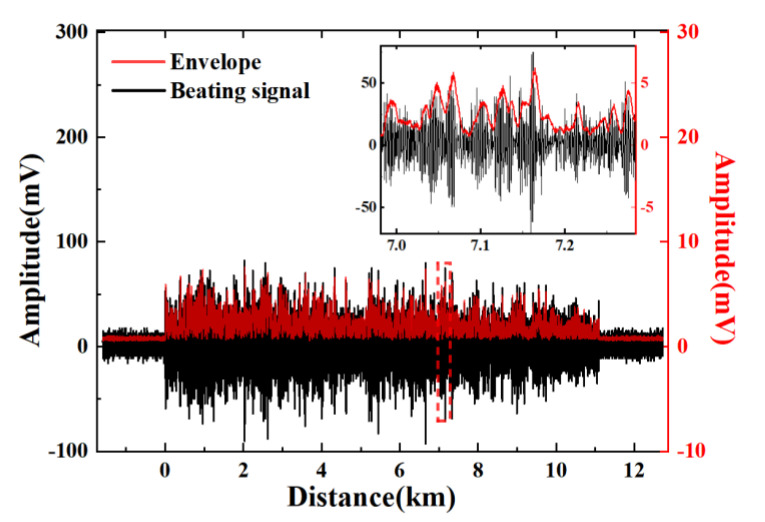
The waveforms of the beating signal and its envelope.

Figure 5 shows the original waveform of the beating signal in black and the amplified signal after the envelope detector in red. A rectified envelope signal is converted from the high-frequency AC beating signal. The envelope signal fits closely to the beating signal. In this transformation, a part of the high-frequency information is lost irreversibly. For the processed signal, the voltage of the noise floor is pulled down to a low level while the intensity of the extracted envelope signal attenuates.

**Figure 6:**
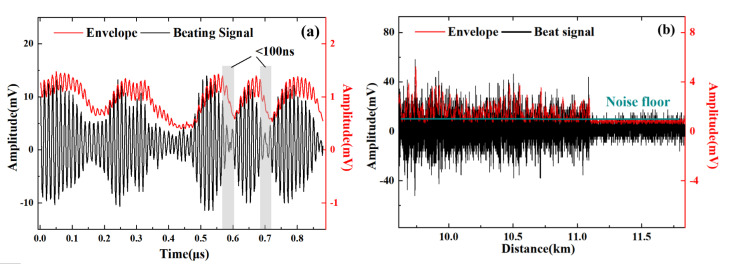
Envelope signal validation for the situations of (**a**) diagonal distortion and (**b**) bottom-cut distortion.

In Figure 6a, there are observable beating signals as carrier waves in the black waveform, and the red line (the signal after the envelope detector) lags a little behind this. It is mainly due to the consequence of the low-pass filter. The frequency of the carrier is 80 MHz, consistent with the frequency shift of the AOM in this experiment.

The red signal fits well with the peaks and troughs of the beating signal, which achieves the goal of extracting the envelope of the beating signal. This preliminary result indicates that no diagonal distortion has occurred with our envelope detector.

An estimated value of the shortest falling edge is obtained from the gray rectangle in Figure 6a. This value indicates the suppression of distortion of the envelope detector. If the period of the envelope signal is shorter than 100 ns, the response frequency of the envelope detector would be greater than 10 MHz. This means that the conditions based on the tested envelope detector circuit are reliable for the requirement of a 10 m spatial resolution. Further experiments are shown in the following sections.

Bottom-cut distortion could occur when a low-intensity beating signal is processed by the envelope detector. This phenomenon is mainly caused by the DC voltage charged on the output capacitor. An ideal situation is that the bottom-cut distortion occurs only in the fading region, where fading noise is dominating to the locating error. Figure 6b shows that the bottom-cut distortion generally occurs below the noise floor in the experiment.

Diagonal distortion and bottom-cut distortion are the main reasons for the large signal’s envelope detecting process. The distortion is acceptable for the following vibration locating experiments based on the analysis.

### 3.3. Effect of Different Carrier Frequencies

Due to the constraints of the hardware, it is difficult to generate carriers with continuous frequency variation. Two situations are compared in this paper for qualitative analysis: an AOM with an 80 MHz frequency shift and another with 200 MHz.

For a conventional coherent Φ-OTDR demodulation system with the condition of a limited sampling rate, the high beating frequency leads to the deterioration of signal quality. Meanwhile, a high carrier frequency contributes to separate the envelope from the electrical beating signal which is collected by a balanced photodetector.

**Figure 7:**
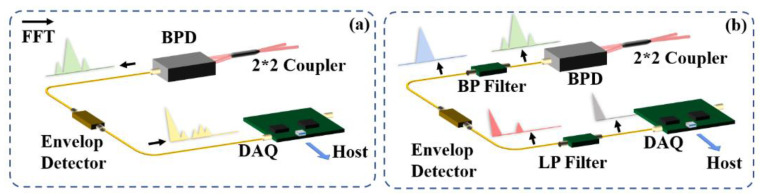
Modified envelope detector scheme for coherent Φ-OTDR. Envelope signal detection based on (**a**) original scheme and (**b**) improved scheme.

For the proposed demodulation scheme in this paper, the envelope detector has different attenuations for different frequency signals. Thus, the noise inside the different carrier bands and broadband white noise can be suppressed to different extents after the envelope detector circuit. In order to improve the quality of the envelope signal, a modified frequency selection processing is applied. A band-pass filter is used to filter out-of-band noise. In addition, an extra low-pass filter is added to suppress high-frequency noise. Most of the high-order sideband components would be eliminated by the filter. The improved settings are shown in Figure 7.

Some defects in the previous pre-experiments are obvious, according to the jagged waveform. When the system obtains the envelope signal from the beating signal, it retains too many high-frequency components, shown as ripples in the red lines in Figure 6a. These noises will deteriorate the signal and reduce the SNR of the system as the spectrum shown in Figure 7a. Therefore, there is an additional low-pass (LP) filter behind the detector circuit. In order to optimize the signal quality, a band-pass (BP) filter is added before the detector to improve the signal quality shown in Figure 7b. In addition, we amplify the signal before entering the detector circuit, which effectively compensates the signal attenuation after the envelope detector.

**Figure 8:**
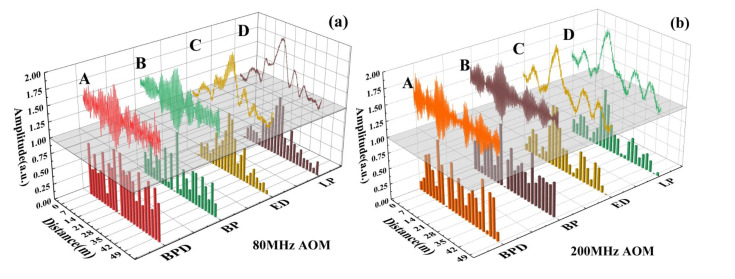
The signals after different processing stages behind the AOMs with frequency shifts of (**a**) 80 MHz and (**b**) 200 MHz.

In Figure 8, waveform A stands for the signal after BPD. Waveform B stands for the signal after band-pass (BP) filter, removing most out-of-band noise. Waveform C stands for the signal after the envelope detector (abbreviated as ED in Figure 8), and waveform D stands for the signal after the low-pass filter to eliminate high-frequency noise and prevent overlap in the frequency domain. The origin signal is shown over the gray XY horizontal plane, and the discrete digital signal is shown below the horizontal plane with a sampling rate of 50 MSps. All the traces have been normalized to fit the coordinate system and compare with each other. In Figure 8a, the envelope detector circuit works in a same way. Nevertheless, the roughly processed envelope signal contains many components with a high-frequency harmonic, such as waveform C; meanwhile, it is generally acceptable for the waveform C with a higher frequency of carrier band in Figure 8b. Finally, a low-pass filter of 40 MHz is set to eliminate the noise and get waveform D. These data can contain the envelope of the beating signal completely without the interference of a high-frequency carrier.

## 4. Results and Discussion

### 4.1. Spatial Resolution Analysis with Different Sampling Rates

Under the limitation of a pulse width of 100 ns, the best result of spatial resolution is up to 10 m. In order to collect the vibration information from the optical fiber per 10 m without distortion, sampling points should be set every 100 ns in the time axis, at least. Based on this condition, the reciprocal pulse width is the minimum requirement of the bandwidth, 10 MSps (1/100 ns) is required at least.

A sinusoidal signal of 1 kHz is exerted to the PZT with a 1.5 m single mode fiber wound put at 10,146 m, and the actual vibration position could be approximately 10,147 m. The sensing data of the envelope signal are collected via different sampling rates. Then, we use the moving differential algorithm to get the vibration position. The differential interval is set to 2.

**Figure 9:**
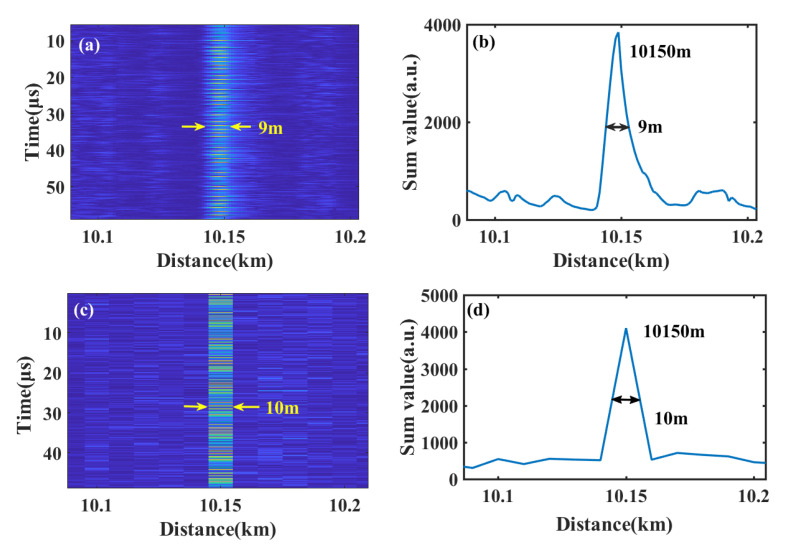
The moving differential results under sampling rates of (**a**) 100 MSps and (**c**) 10 MSps; the sum results of moving differential algorithm under sampling rates of (**b**) 100 MSps and (**d**) 10 MSps.

**Figure 10:**
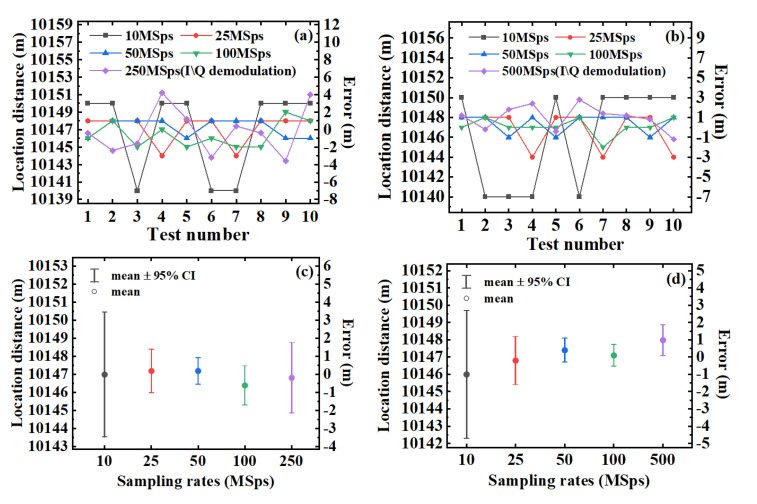
Results of vibration locating under different sampling rates and schemes using (**a**) the AOM of 80 MHz and (**b**) the AOM of 200 MHz; the mean value and mean value ± 95% confidence interval (CI) for the results of vibration locating using (**c**) the AOM of 80 MHz and (**d**) the AOM of 200 MHz.

In Figure 9, (a) and (b) represent the vibration location results under 100 MSps, (c) and (d) are the results under 10 MSps. The demodulation results are both 10,150 m in Figure 9b,d. The full width at half maximum (FWHM) of the sum values are calculated to be 9 m (under 100 MSps) or 10 m (under 10 MSps). These values are within the spatial resolution of 10 m in theory. More experiment results under 10 MSps, 25 MSps, 50 MSps, 100 MSps, 250 MSps and 500 MSps are shown in Figure 10.

In Figure 10, the signal traces are sampled by a DAQ, whose sampling rate is set from 10 MSps to 100 MSps in the vibration locating scheme based on the envelope detector. In the meantime, the sampling rates are set to 250 MSps (using the AOM of 80 MHz) and 500 MSps (using the AOM of 200 MHz) for the traditional I/Q demodulation scheme.

Figure 10a,b show the repeatability results with 10 testing groups directly. The curves reflect the fluctuation of locating errors under different sampling rates and different demodulation schemes. In addition, we calculate the mean values and the 95% confidence intervals of these different curves, which are shown in Figure 10c,d.

It can be seen that similar results of vibration position are obtained with both vibration locating schemes. The errors of locating results are within the allowable range of 10 m spatial resolution. In the vibration locating scheme based on envelope detector, the locating results show that the lowest acceptable sampling rate reaches 10 MSps to meet the spatial resolution of 10 m in Figure 10.

### 4.2. SNR Analysis in Vibration Locating Experiment

In this vibration locating experiment, a series of pulses with a repetition frequency of 8 kHz and a pulse width of 100 ns is used to trigger the 80 MHz AOM and 200 MHz AOM. The proposed scheme based on an envelope detector and traditional I/Q demodulation scheme are applied for vibration locating.

**Figure 11:**
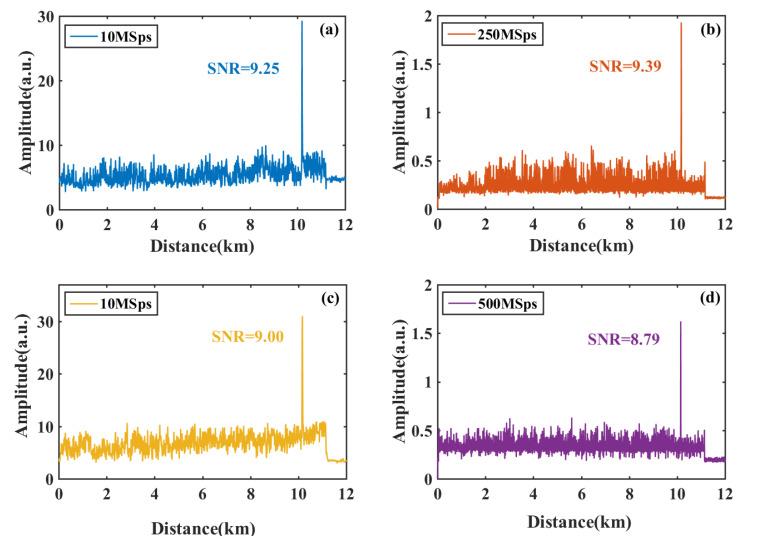
Vibration locating results with 80 MHz AOM using (**a**) envelope detector scheme under 10 MSps and (**b**) I/Q demodulation scheme under 250 MSps; results with 200 MHz AOM using (**c**) envelope detector scheme under 10 MSps and (**d**) I/Q demodulation scheme under 500 MSps.

The moving differential algorithm is applied to get the vibration locating results. Then, the differential results are summed to improve the SNR. Here, the SNR is defined as $20*\log\left(\frac{V_{signal}}{V_{noise}}\right)$, where $V_{signal}$ stands for the peak value of the signal, and $V_{noise}$ stands for the peak value of the noise. The simulated vibration is set to a sinusoidal signal of 1 kHz on PZT at 10,147 m. The results of the accumulation process are shown in Figure 11.

In Figure 11b,d, the original beating signal is acquired under high sampling rates to avoid the signal distortion. I and Q components are obtained by multiplying two sinusoidal sequences with a phase difference of 90 degrees, respectively. Combined with the results of the previous process, we obtain the RBS amplitude through a low-pass filter. However, in Figure 11a,c, envelope signals are acquired under the sampling rate of 10 MSps.

The SNR of the envelope detector scheme is 0.14 dB smaller than the I/Q demodulation scheme in the experiment with the 80 MHz AOM. The SNR of the envelope detector scheme is 0.21 dB higher than the I/Q demodulation scheme in the experiment using a 200 MHz AOM.

Apparently, there is no significant difference in the SNR between these two schemes, according to Figure 11. The results show that the vibration locating performance of the envelope detector scheme (shown in Figure 11a,c) is close to that of the traditional I/Q demodulation scheme (shown in Figure 11b,d).

**Figure 12:**
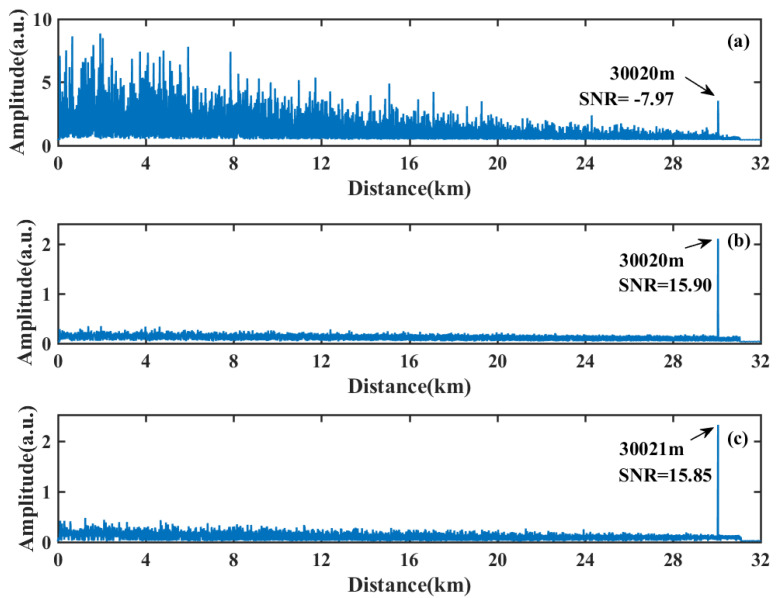
Vibration locating results over 31 km with (**a**) direct detection scheme, (**b**) envelope detector and moving differential algorithm scheme, and (**c**) I/Q demodulation scheme.

In order to realize a longer sensing distance with a high SNR, a laser with a narrow linewidth of 100 Hz is applied, with the pulse repetition frequency of 3 kHz and the pulse width of 100 ns. The tested optical fiber is set to 31 km, a 5 Vpp sinusoidal signal at 1.5 kHz is exerted on the PZT at 30,017 m. The vibration locating results based on three schemes are shown in Figure 12.

The summed results of the moving differential process are based on different sampling rates. The acquisition requirement of the direct detection scheme is 10 MSps, which equals the scheme base on the envelope detector. In the coherent detection schemes (Figure 12b,c), the AOM of 80 MHz is applied. The sampling rate of the I/Q demodulation scheme reaches 250 MSps.

The vibration locating peak is confused with many ghost peaks at the beginning of the optical fiber for the direct detection scheme, which is shown in Figure 12a. These coherent detection results based on the envelope detector scheme (Figure 12b) and I/Q demodulation scheme (Figure 12c) are proximal, which reach the SNR at 15.90 dB and 15.85 dB, respectively. This means that in long-distance sensing applications, the scheme based on the envelope detector can be a low sampling rate substitute without the loss of the SNR and spatial resolution.

## 5. Conclusions

In this paper, the low sampling rate coherent Φ-OTDR based on the envelope extraction scheme gets a significant algorithmic simplification. It can effectively convert the high-frequency beating signal into the low-frequency envelope signal. Therefore, a sharply reduced requirement of sampling rate can be realized by the envelope extraction scheme. Furthermore, a vibration locating process can be largely simplified with the help of a basic moving differential algorithm of the envelope signal, avoiding the heavy computing consumption. With the envelope extraction scheme, vibration locating results over 30 km with a high spatial resolution of 10 m can be obtained under a sampling rate of 10 MSps. After the performance comparison, the SNR and the spatial resolution of the envelope extraction scheme are close to that of the I/Q demodulation scheme under the sampling rate of 500 MSps (using the AOM of 200 MHz) or 250 MSps (using the AOM of 80 MHz). This improved vibration locating method with reduced sampling rate can be a useful method for the real vibration monitoring applications.

## Data Availability

All original data, vibration data and code will be made available on request to the correspondent author’s email with appropriate justification.
